# Immunohistochemical quantification of the cobalamin transport protein, cell surface receptor and Ki-67 in naturally occurring canine and feline malignant tumors and in adjacent normal tissues

**DOI:** 10.18632/oncotarget.3206

**Published:** 2014-12-11

**Authors:** Annette M. Sysel, Victor E. Valli, Joseph A. Bauer

**Affiliations:** ^1^ Bauer Research Foundation, Akron, Ohio, USA; ^2^ VDx Veterinary Pathology Services, Davis, California, USA

**Keywords:** transcobalamin II, Ki-67, canine, feline, tumors

## Abstract

Cancer cells have an obligate need for cobalamin (vitamin B_12_) to enable DNA synthesis necessary for cellular replication. This study quantified the immunohistochemical expression of the cobalamin transport protein (transcobalamin II; TCII), cell surface receptor (transcobalamin II-R; TCII-R) and proliferation protein (Ki-67) in naturally occurring canine and feline malignant tumors, and compared these results to expression in corresponding adjacent normal tissues. All malignant tumor tissues stained positively for TCII, TCII-R and Ki-67 proteins; expression varied both within and between tumor types. Expression of TCII, TCII-R and Ki-67 was significantly higher in malignant tumor tissues than in corresponding adjacent normal tissues in both species. There was a strong correlation between TCII and TCII-R expression, and a modest correlation between TCII-R and Ki-67 expression in both species; a modest association between TCII and Ki-67 expression was present in canine tissues only. These results demonstrate a quantifiable, synchronous up-regulation of TCII and TCII-R expression by proliferating canine and feline malignant tumors. The potential to utilize these proteins as biomarkers to identify neoplastic tissues, streamline therapeutic options, evaluate response to anti-tumor therapy and monitor for recurrent disease has important implications in the advancement of cancer management for both human and companion animal patients.

## INTRODUCTION

As the ‘one treatment fits all’ paradigm disappears from clinical cancer medicine, there is an increasing demand for the discovery and development of new cancer biomarkers to facilitate the transition to individualized patient care. Biomarkers offer the potential for improved diagnostic and therapeutic efficacy and reduced toxicity compared to traditional tumor management options [1, 2]. The ideal cancer biomarker is one which can be used to diagnose disease at an early stage, tailor therapy specific to each patient, monitor response to therapy, recognize potential therapeutic toxicities, define prognosis and monitor disease progression or recurrence [3-5]. Given that the genetic and molecular alterations involved in cancer ultimately result in the aberrant expression of protein products [6], and that increased or decreased protein production directly influences molecular pathways in both normal and malignant cells [7], proteins continue to serve as optimal cancer biomarkers. Several protein tumor markers are currently approved for use in clinical practice, and some of the most common include human epidermal growth factor 2 (HER2/neu), estrogen receptor (ER), progesterone receptor (PR), prostate-specific antigen (PSA) and receptor tyrosine kinase (cKit) [8].

Cobalamin (Cbl; vitamin B_12_) is an essential micronutrient that plays an important role in the differentiation, proliferation and metabolic stability of cells [9]. All living cells have an obligate requirement for Cbl, which is crucial for the methylation process associated with DNA synthesis [10-12]. The transcobalamin II transport protein (TCII) and cell surface receptor (TCII-R) are essential proteins involved in Cbl transport and uptake, and are expressed in all mammalian species [13]. Upon absorption into the bloodstream, circulating Cbl is immediately bound to TCII, which is produced by many cell types, including endothelial cells [9, 12, 14, 15]. The TCII-Cbl complex travels to the cell membrane surface and undergoes receptor-mediated endocytosis via TCII-R, which specifically recognizes TCII-bound Cbl [16].

Active tumor cells undergoing differentiation and proliferation have an especially high requirement for Cbl to support rapid and continued cell division [12, 17-19]. Increased demand for Cbl is believed to be supplied by two basic mechanisms: i) tumor cells themselves produce TCII that is then able to scavenge, bind, transport and deliver Cbl to the cells [20, 21], and ii) cell surface TCII-R levels are up-regulated to import more Cbl when the cells are in a proliferative mode [22, 23]. Increased quantities of TCII and TCII-R proteins produced by the tumor itself may therefore be important quantifiable markers of tumor biologic activity.

Recently we developed a standardized, objective immunohistochemical (IHC) method to quantify expression of TCII, TCII-R and Ki-67, a well-established protein marker of cellular proliferation, in a variety of human malignant tumor xenograft tissues [24]. We were able to demonstrate measurable levels of TCII, TCII-R and Ki-67 expression in all xenograft tumor tissues [24]. To our knowledge, TCII and TCII-R expression has not been measured in non-xenograft, malignant tumor tissues. The aim of the present study was to quantify expression of TCII and TCII-R in a variety of naturally occurring, readily available canine and feline malignant tumors, to compare expression of these proteins between tumors and their corresponding adjacent normal tissues, and to determine whether tumor expression of TCII and TCII-R demonstrates a correlation with tumor proliferation status as assessed by measurement of Ki-67 expression. Up-regulation of TCII and TCII-R proteins in tumor tissues may validate their potential use as biomarkers for identification and monitoring of malignant tissues and for selection and evaluation of Cbl-based anti-tumor therapies.

## RESULTS

### Canine study population

Of the thirty dogs whose archived tissues were used in this study, 20 dogs were purebreds, and 10 were of mixed breed. Purebred breeds included Alaskan Malamute, Australian Cattle Dog, Australian Shepherd, Beagle (n = 2), Chihuahua, Dachshund, German Shepherd, Golden Retriever (n = 2), Greater Swiss Mountain Dog, Labrador Retriever, Malinois, Portuguese Water Dog, Poodle, Rottweiler (n = 2), Scottish terrier, Shih Tzu and West Highland White Terrier. Median age of dogs was 10 years. Of the 30 dogs, 16 were neutered males and 12 were spayed females; sex was not recorded for 2 dogs.

Three cases each of ten different canine malignant tumor types were evaluated, including:

(1) anal gland adenocarcinoma; (2) digital squamous cell carcinoma; (3) soft tissue sarcoma; (4) splenic hemangiosarcoma; (5) lymphoma; (6) melanoma; (7) appendicular osteosarcoma; (8) prostatic carcinoma; (9) thyroid carcinoma and (10) urinary bladder transitional cell carcinoma. The soft tissue sarcoma tumor group was represented by a thoracic soft tissue sarcoma (case no. 7), a soft tissue sarcoma over the elbow (case no. 8) and a peripheral nerve sheath tumor (case no. 9). Tumors in the lymphoma group originated from the lip in one dog (T-cell epitheliotropic tumor, case no. 13) and from the submandibular lymph nodes in two dogs (large B-cell centroblastic tumor, case no. 14; and T-cell small cell tumor, case no. 15). Of the melanomas, one tumor was cutaneous (case no. 16) and the other two were oral, originating from the tongue in one dog (case no. 17) and the mandible in the other (case no. 18). All osteosarcomas were humeral in origin, with two originating from the proximal humerus (case nos. 19, 21) and one from the distal humerus (case no. 20).

### Canine immunohistochemical staining results

**Fig.1:**
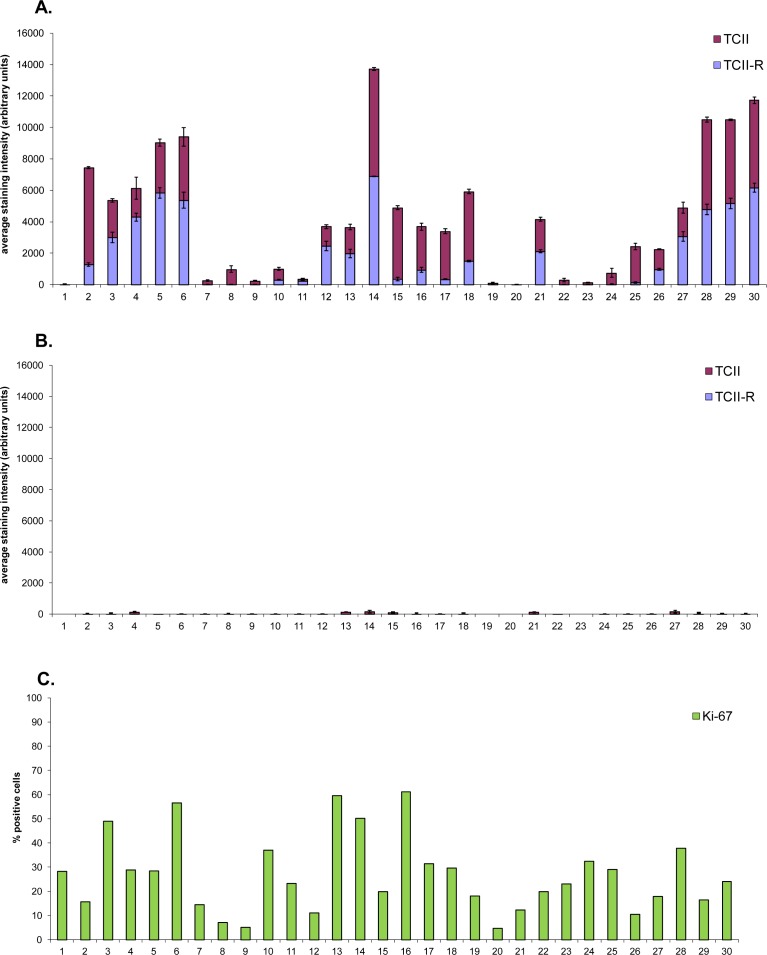

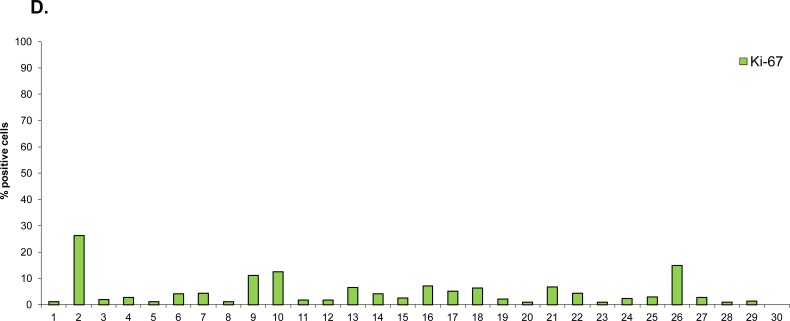
Graphical representation of TCII, TCII-R and Ki-67 expression in canine malignant tumor tissues and corresponding adjacent normal tissues Tissue samples were immunohistochemically stained for TCII (TCN2 antibody), TCII-R (CD320 antibody) and Ki-67 (MIB-1 antibody). TCII and TCII-R data are represented as mean +/− SEM. (A) TCII and TCII-R expression in canine malignant tumor tissues. (B) TCII and TCII-R expression in corresponding adjacent normal canine tissues. (C) Ki-67 expression in canine malignant tumor tissues. (D) Ki-67 expression in corresponding adjacent normal canine tissues. X-axis case identification includes: (1) anal gland adenocarcinoma 11090371, (2) anal gland adenocarcinoma 11091836, (3) anal gland adenocarcinoma 11101247, (4) digital squamous cell carcinoma 11110453, (5) digital squamous cell carcinoma 11120632, (6) digital squamous cell carcinoma 11120413, (7) fibrosarcoma 11120641, (8) fibrosarcoma 11120720, (9) fibrosarcoma 11120721, (10) splenic hemangiosarcoma 11070302, (11) splenic hemangiosarcoma 11101549, (12) splenic hemangiosarcoma 11101711, (13) lymphoma 11111603, (14) lymphoma 11120588, (15) lymphoma 11120684, (16) melanoma (cutaneous) 11070597, (17) melanoma (oral) 11061907, (18) melanoma (oral) 11070304, (19) osteosarcoma 11061788, (20) osteosarcoma 11061908, (21) osteosarcoma 11070395, (22) prostatic carcinoma 11032215, (23) prostatic carcinoma 11041574, (24) prostatic carcinoma 11071912, (25) thyroid carcinoma 11110836, (26) thyroid carcinoma 11111199, (27) thyroid carcinoma 11111344, (28) urinary bladder transitional cell carcinoma 10100415, (29) urinary bladder transitional cell carcinoma 11121036, (30) urinary bladder transitional cell carcinoma 11120309.

**Fig.2:**
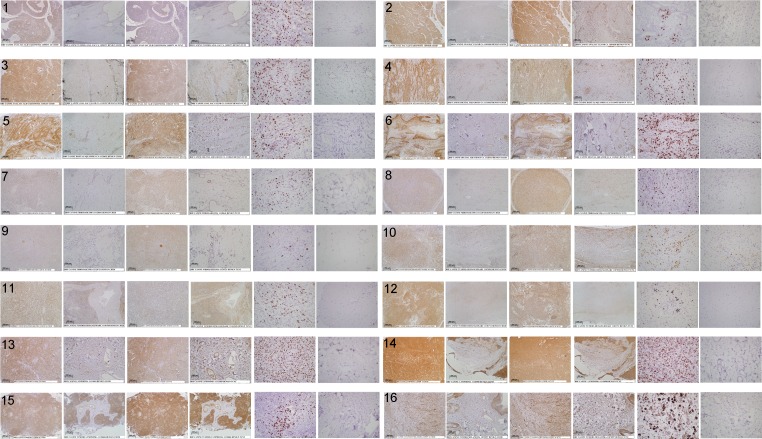

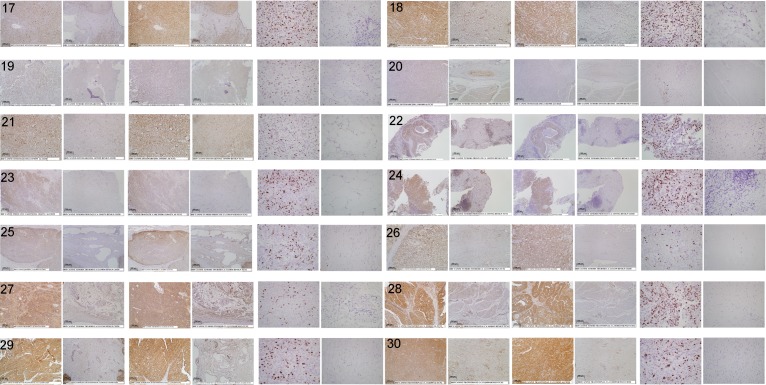
Digital images of stained canine tissue sections Tissue samples were immunohistochemically stained for TCII (TCN2 antibody, 10x objective), TCII-R (CD320 antibody, 10x objective) and Ki-67 (MIB-1 antibody, 40x objective). Tissue sections for each case are shown from left to right as follows: TCII tumor, TCII adjacent normal, TCII-R tumor, TCII-R adjacent normal, Ki-67 tumor, Ki-67 adjacent normal. Refer to Figure 1 legend for x-axis case identification. High resolution images of case 30 are presented in Supplemental data.

Immunohistochemical staining values for canine tissues are summarized in Table 1 and illustrated graphically in Figure 1; digital images of all stained slides are shown in Figure 2.

**Table 1:** Average TCII, TCII-R and Ki-67 staining values for canine and feline tumor tissues and corresponding adjacent normal tissues

	TCII ^tumor^ (AU)	TCII ^adj normal^ (AU)	TCII-R ^tumor^ (AU)	TCII-R ^adj normal^ (AU)	Ki-67 ^tumor^ (%)	Ki-67 ^adj normal^ (%)
**CANINE**						
anal gland adenocarcinoma	2849	29	1431	4	31	10
digital squamous cell carcinoma	3016	48	5170	0.5	38	3
fibrosarcoma	478	15	13	5	9	6
hemangiosarcoma	689	6	1003	2	24	5
lymphoma	4339	130	3079	10	43	5
melanoma	3412	47	924	3	41	6
osteosarcoma	706	43	721	1	12	3
prostatic carcinoma	361	8	23	0	25	3
thyroid carcinoma	1794	48	1391	16	19	7
bladder transitional cell carcinoma	5526	51	5374	4	26	1
**FELINE**						
biliary carcinoma	6055	124	5040	0.7	42	4
dermal carcinoma	2922	10	4039	0	43	10
fibrosarcoma - vaccinal	5785	19	2245	0.5	34	2
intestinal adenocarcinoma	2303	5	2532	2	49	3
intestinal lymphoma	2806	44	2076	3	71	3
intestinal mast cell tumor	929	0.3	363	0	3	3
mammary adenocarcinoma	5548	0.7	5263	0.2	38	6
nodal lymphoma	5897	0.1	3478	0	30	8
oral squamous cell carcinoma	6246	74	5228	0.2	35	5
soft tissue sarcomas	752	0.5	189	0.2	16	4
plenic mast cell tumor	2120	67	1270	0	16	5
bladder transitional cell carcinoma	5425	14	6071	3	40	2

^tumor^ = tumor tissue samples; ^adj normal^ = corresponding adjacent normal tissue samples; AU = arbitrary staining units; % = percentage of positively stained cells

All canine malignant tumors stained positively for TCII, TCII-R and Ki-67, with the exception of one osteosarcoma case which did not express staining for TCII. There was no significant correlation between TCII, TCII-R or Ki-67 expression and breed, age, weight or sex. Immunohistochemical staining values for TCII, TCII-R and Ki-67 were of similar value within some tumor tissue types, and were more varied within others. Overall average/median staining values of canine malignant tumor tissues were 2317/1824 (TCII), 1913/955 (TCII-R) and 27/23% (Ki-67); range of staining values was 0-6817 (TCII), 3-6897 (TCII-R) and 4-61% (Ki-67).

### TCII, TCII-R and Ki-67 staining intensity among 10 common canine malignant tumor types

Compared to average TCII and TCII-R staining values, canine malignant tumors with the highest degree of TCII and TCII-R staining included urinary bladder transitional cell carcinoma, digital squamous cell carcinoma and lymphoma. Tumors with a moderate degree of TCII and TCII-R staining included melanoma, anal gland adenocarcinoma and thyroid carcinoma. Tumors with the lowest TCII and TCII-R staining intensity were hemangiosarcoma, osteosarcoma, prostatic carcinoma and soft tissue sarcoma. In the osteosarcoma cases, staining intensity was influenced by the amount of bone present in the sample (i.e. tumors with more osseous tissue in the sample exhibited less staining). Compared to the average Ki-67 staining value, the most proliferative canine malignant tumors were lymphoma, melanoma and digital squamous cell carcinoma, and the least proliferative included osteosarcoma and soft tissue sarcoma.

### TCII, TCII-R and Ki-67 expression was up-regulated in canine malignant tumors

Adjacent normal tissues expressed significantly less staining for TCII, TCII-R and Ki-67. Overall average/median staining values of adjacent normal tissues were 42/12 (TCII), 5/0.5 (TCII-R) and 5/3% (Ki-67); range of staining values was 0-151 (TCII), 0-47 (TCII-R) and 0-26% (Ki-67). For every case, there was a statistically significant difference between TCII, TCII-R and Ki-67 expression in malignant tumor tissue compared to that in the corresponding adjacent normal tissue (TCII: p<0.000001; TCII-R: p<0.0001 ; Ki-67: p< 0.00000001).

### Canine tissues stained more highly for TCII than for TCII-R

TCII staining values were higher than those observed for TCII-R in the majority of both malignant tumor and adjacent normal tissues: 60% of malignant tissues (median difference of 5.5 times greater) and 80% of normal tissues (median difference of 9.5 times greater) had higher TCII staining values compared to TCII-R staining values.

### TCII and TCII-R expression was correlated in canine malignant tumors

There was a statistically significant correlation between TCII and TCII-R expression in all malignant tumor tissues (Spearman rank correlation coefficient value; *r*_s_: 0.78; p < 0.05). There was also a modest correlation between TCII and Ki-67 expression (*r*_s_: 0.40, p< 0.05) and between TCII-R and Ki-67 expression (*r*_s_: 0.41, p< 0.05 ) in all malignant tumor tissues.

### Feline study population

Of the thirty-six cats whose archived tissues were used in this study, breeds included Persian (n = 1), Siamese (n = 2), domestic longhair (n = 5) and domestic shorthair (n = 28). Median age of cats was 13 years. Of the 36 cats, 2 were intact females, 15 were spayed females and 19 were neutered males.

Three cases each of twelve different feline tumor types were evaluated and included: (1) biliary carcinoma; (2) dermal carcinoma; (3) vaccine-associated fibrosarcoma; (4) intestinal adenocarcinoma; (5) intestinal lymphoma; (6) intestinal mast cell tumor; (7) mammary adenocarcinoma; (8) nodal lymphoma; (9) oral squamous cell carcinoma; (10) soft tissue sarcoma; (11) splenic mast cell tumor and (12) urinary bladder transitional cell carcinoma. Of these tumors, all dermal carcinomas were histologically identified as Bowenoid carcinomas, and originated from the left shoulder in one cat (case no. 4), from the left temporal region in one cat (case no. 5), and from the left side of the forehead/ventral abdomen in one cat (case no. 6). All three vaccine-associated fibrosarcomas were located in the right thigh/flank region. Intestinal adenocarcinomas originated from the ileocecocolic junction (case no. 11) and the ileocecal junction (case no. 12); location was not specified in the third cat (case no. 10). Intestinal lymphomas included a large-cell jejunal tumor (case no. 13), a large-cell tumor (unspecified site, case no. 14) and an intermediate-cell colonic tumor (case no. 15). Two intestinal mast cell tumors were jejunal in origin (case nos. 16, 18); location was not specified in the third cat (case no. 17). All nodal lymphomas were classified as T-cell rich large B-cell tumors, and originated from the submandibular region in two cats (case nos. 22, 23) and from the retropharyngeal region in one cat (case no. 24). Soft tissue sarcomas included a subcutaneous sarcoma over the right shoulder (case no. 28), a sarcoma over the left shoulder (case no. 29), and a neurofibrosarcoma over the right shoulder (case no. 30).

### Feline immunohistochemical staining results

**Fig.3:**
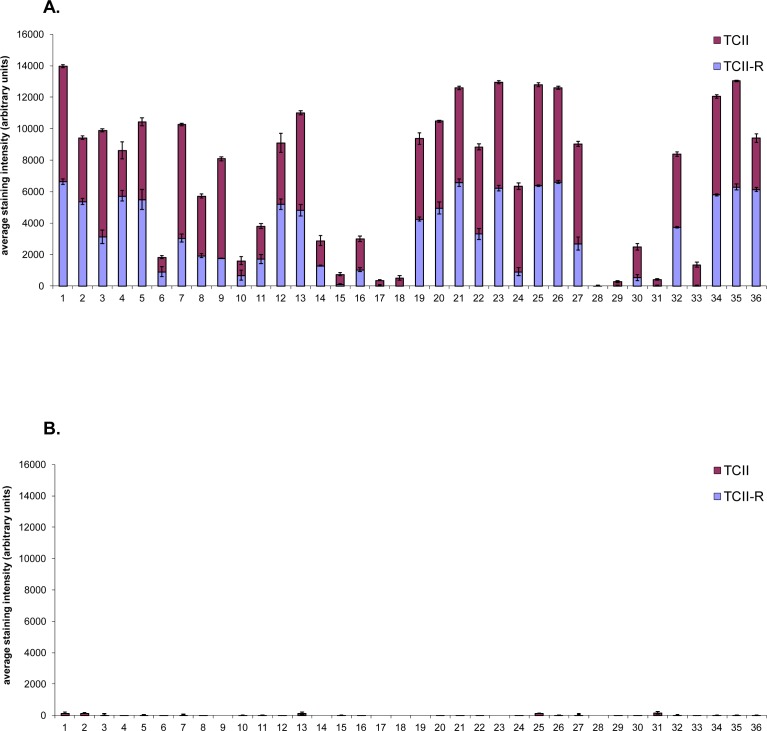

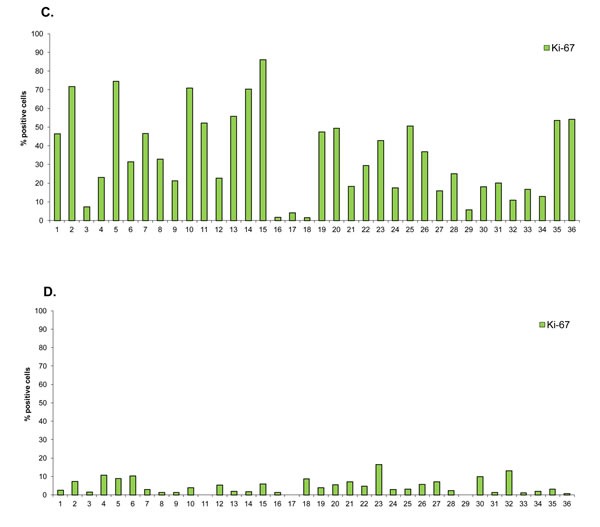
Graphical representation of TCII, TCII-R and Ki-67 expression in feline malignant tumor tissues and corresponding adjacent normal tissues Tissue samples were immunohistochemically stained for TCII (TCN2 antibody), TCII-R (CD320 antibody) and Ki-67 (MIB-1 antibody). TCII and TCII-R data are represented as mean +/− SEM. (A) TCII and TCII-R expression in feline malignant tumor tissues. (B) TCII and TCII-R expression in corresponding adjacent normal feline tissues. (C) Ki-67 expression in feline malignant tumor tissues. (D) Ki-67 expression in corresponding adjacent normal feline tissues. X-axis case identification includes: (1) biliary carcinoma 10021255, (2) biliary carcinoma 10101918, (3) biliary carcinoma 10121242, (4) dermal carcinoma 10071506, (5) dermal carcinoma 11030645, (6) dermal carcinoma 11111180, (7) fibrosarcoma - vaccine 10051260, (8) fibrosarcoma - vaccine 11120550, (9) fibrosarcoma - vaccine 9070268, (10) intestinal adenocarcinoma 11100237, (11) intestinal adenocarcinoma 11101952, (12) intestinal adenocarcinoma 11120080, (13) intestinal lymphoma 11101365, (14) intestinal lymphoma 10120094, (15) intestinal lymphoma 11120834, (16) intestinal mast cell tumor 11071015, (17) intestinal mast cell tumor 11080200, (18) intestinal mast cell tumor 11111192, (19) mammary adenocarcinoma 11051222, (20) mammary adenocarcinoma 11051232, (21) mammary adenocarcinoma 11071104, (22) nodal lymphoma 11111342, (23) nodal lymphoma 11111667, (24) nodal lymphoma 11112001, (25) oral squamous cell carcinoma 11110315, (26) oral squamous cell carcinoma 11120118, (27) oral squamous cell carcinoma 11120336, (28) sarcoma 11041581, (29) soft tissue sarcoma 11011745, (30) soft tissue sarcoma 11091746, (31) splenic mast cell tumor 11031700, (32) splenic mast cell tumor 11050011, (33) splenic mast cell tumor 11050097, (34) urinary bladder transitional cell carcinoma 10060321, (35) urinary bladder transitional cell carcinoma 11011306, (36) urinary bladder transitional cell carcinoma 11041886.

**Fig.4:**
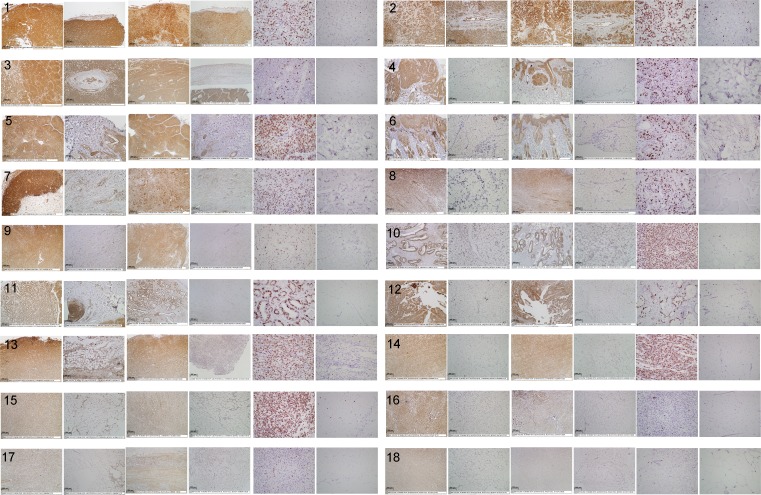

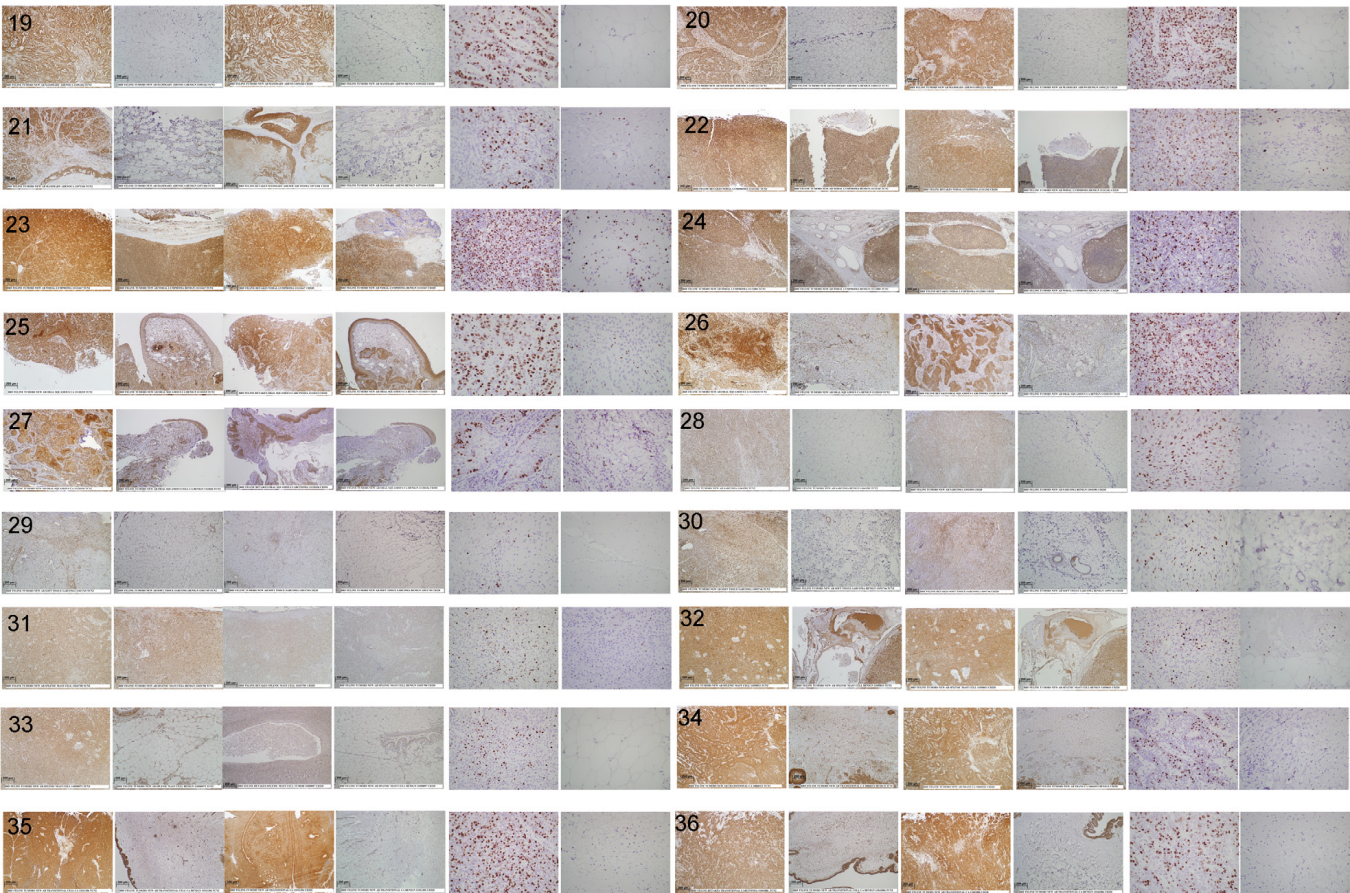
Digital images of stained feline tissue sections Tissue samples were immunohistochemically stained for TCII (TCN2 antibody, 10x objective), TCII-R (CD320 antibody, 10x objective) and Ki-67 (MIB-1 antibody, 40x objective). Tissue sections for each case are shown from left to right as follows: TCII tumor, TCII adjacent normal, TCII-R tumor, TCII-R adjacent normal, Ki-67 tumor, Ki-67 adjacent normal. Refer to Figure 3 legend for x-axis case identification. High resolution images of case 36 are presented in Supplemental data.

Immunohistochemical staining values for feline tissues are summarized in Table 1 and illustrated graphically in Figure 3; digital images of all stained slides are shown in Figure 4.

All feline malignant tumors stained positively for TCII, TCII-R and Ki-67, with the exception of one intestinal mast cell tumor case which did not express staining for TCII-R. There was no significant correlation between TCII, TCII-R or Ki-67 expression and breed, age or sex. Similar to canine tissues, immunohistochemical staining values for TCII, TCII-R and Ki-67 were relatively consistent within some tumor tissue types, and were more varied within others. Overall average/median staining values of feline malignant tumor tissues were 3899/4352 (TCII), 3149/3092 (TCII-R) and 35/31% (Ki-67); range of staining values was 36-7346 (TCII), 0-6622 (TCII-R) and 2-86% (Ki-67). Average staining values for TCII, TCII-R and Ki-67 were significantly higher in feline malignant tumor tissues than in canine malignant tumor tissues (p<0.05).

### TCII, TCII-R and Ki-67 staining intensity among 12 common feline malignant tumor types

Compared to average TCII and TCII-R staining values, feline malignant tumors with the highest degree of TCII and TCII-R staining included urinary bladder transitional cell carcinoma, oral squamous cell carcinoma, biliary carcinoma, mammary adenocarcinoma and nodal lymphoma. Tumors with a moderate degree of TCII and TCII-R staining included vaccine-associated fibrosarcoma and dermal carcinoma. Tumors with the lowest TCII and TCII-R staining intensity were intestinal lymphoma, intestinal adenocarcinoma, splenic mast cell tumor, intestinal mast cell tumor and soft tissue sarcoma. Compared to the average Ki-67 staining value, the most proliferative feline malignant tumors were intestinal lymphoma and intestinal adenocarcinoma, and the least proliferative included soft tissue sarcoma, splenic mast cell tumor and intestinal mast cell tumor.

### TCII, TCII-R and Ki-67 expression was up-regulated in feline malignant tumors

As in the canine tissues, adjacent normal tissues expressed significantly less staining for TCII, TCII-R and Ki-67. Overall average/median staining values of adjacent normal tissues were 30/1 (TCII), 0.7/0 (TCII-R) and 5/3% (Ki-67); range of staining values was 0-166 (TCII), 0-9 (TCII-R) and 0-17% (Ki-67). For every case, there was a statistically significant difference between TCII, TCII-R and Ki-67 expression in malignant tumor tissue compared to that in corresponding adjacent normal tissue (TCII: p<0.0000000000001; TCII-R: p<0.0000000001; Ki-67: p<0.0000000001).

### Feline tissues stained more highly for TCII than for TCII-R

Similar to canine tissues, TCII staining values were higher than those observed for TCII-R in the majority of both malignant tumor and adjacent normal tissues: 81% of malignant tissues (median difference of 1.9 times greater) and 72% of normal tissues (median difference of 6.5 times greater) had higher TCII staining values compared to TCII-R staining values.

### TCII and TCII-R expression was correlated in feline malignant tumors

There was a statistically significant correlation between TCII and TCII-R expression in all malignant tumor tissues (*r*_s_: 0.76; p < 0.05) which was virtually identical to the correlation observed in canine malignant tumor tissues. There was a modest correlation between TCII-R and Ki-67 expression in all feline malignant tumor tissues (*r*_s_: 0.36, p< 0.05) but not between TCII and Ki-67 expression (*r*_s_: 0.15, p>0.05).

### Comparison of immunohistochemical staining among similar feline malignant tumor types

Among feline malignant tumor types, TCII and TCII-R staining values were significantly higher in feline vaccine-associated fibrosarcomas than in soft tissue sarcomas (TCII: p < 0.0134; TCII-R: p < 0.0098). There were no significant differences in TCII and TCII-R staining between cases of feline intestinal and nodal lymphoma, or between feline intestinal and splenic mast cell tumors; however, Ki-67 staining was significantly higher in feline intestinal lymphomas compared to nodal lymphomas (p < 0.0231) and in splenic mast cell tumors compared to intestinal mast cell tumors (p < 0.0089). TCII and TCII-R, but not Ki-67, staining values were higher in the 2 cases of large cell intestinal lymphoma than in the single case of intermediate cell intestinal lymphoma.

### Comparison of immunohistochemical staining among similar canine and feline malignant tumor types

Three similar tumor types were evaluated in both canine and feline species, and these included urinary bladder transitional cell carcinoma, soft tissue sarcoma and lymphoma. There was no statistically significant difference in TCII, TCII-R or Ki-67 staining values between canine and feline urinary bladder transitional cell carcinomas, between canine and feline soft tissue sarcomas or between canine and feline lymphomas (including both feline nodal and intestinal forms). There was, however, a significant difference in TCII and TCII-R staining values between feline vaccine-associated fibrosarcomas and both canine soft tissue sarcomas (TCII: p<0.0073; TCII-R: p<0.0054) and feline soft tissue sarcomas and (TCII: p< 0.0134; TCII-R: p<0.0098), with higher staining values observed for feline vaccine-associated fibrosarcomas in both species; staining values for Ki-67 were significantly higher in feline vaccine-associated fibrosarcomas compared to canine soft tissue sarcomas (p<0.0352), but not compared to feline soft tissue sarcomas.

## DISCUSSION

Since the original isolation and crystallization of cobalamin in 1948 [25, 26], this study is the first to quantify on a molecular level the expression of the cobalamin transport protein and cell surface receptor in canine and feline malignant tumors. It is also the first study to quantify TCII, TCII-R and Ki-67 protein expression in a wide variety of naturally occurring malignant tumor tissues under standardized IHC staining, imaging and analysis conditions, and to measure the up-regulation of these proteins in comparison to corresponding adjacent normal tissues. Previous studies evaluating Ki-67 expression in malignant tumor tissues have used benign masses [27-29], hyper/dysplastic lesions [27-30], normal tissues from similar areas in different patients [28, 30, 31] or normal tissues from similar areas in same patients [29, 32] as comparative non-malignant tissues, but few studies have evaluated juxtaposed normal peri-tumoral tissues to document significant protein biomarker up-regulation by tumors.

All tissues evaluated in this study originated from formalin-fixed samples obtained from canine and feline patients at the time of surgical tumor resection or biopsy. Processing and staining of all tissues were conducted in the same laboratory, and same lots of primary antibodies were utilized to enable a direct comparative analysis of protein expression between tissues. Digital images of stained slides were obtained by a single pathologist, ensuring consistent representation of region of interest in all tissues. Images were obtained under the same magnifications and light settings to eliminate variables that may affect computer-assisted analysis. Use of color deconvolution and ImageJ plugins for computer-based image analysis permitted the detection of minute areas of immunoreactivity, allowing accurate calculations of positively stained areas. Upper and lower thresholds for histogram analysis of color deconvoluted images were standardized for every image, enabling direct comparison of staining values between tissues.

**Fig.5:**
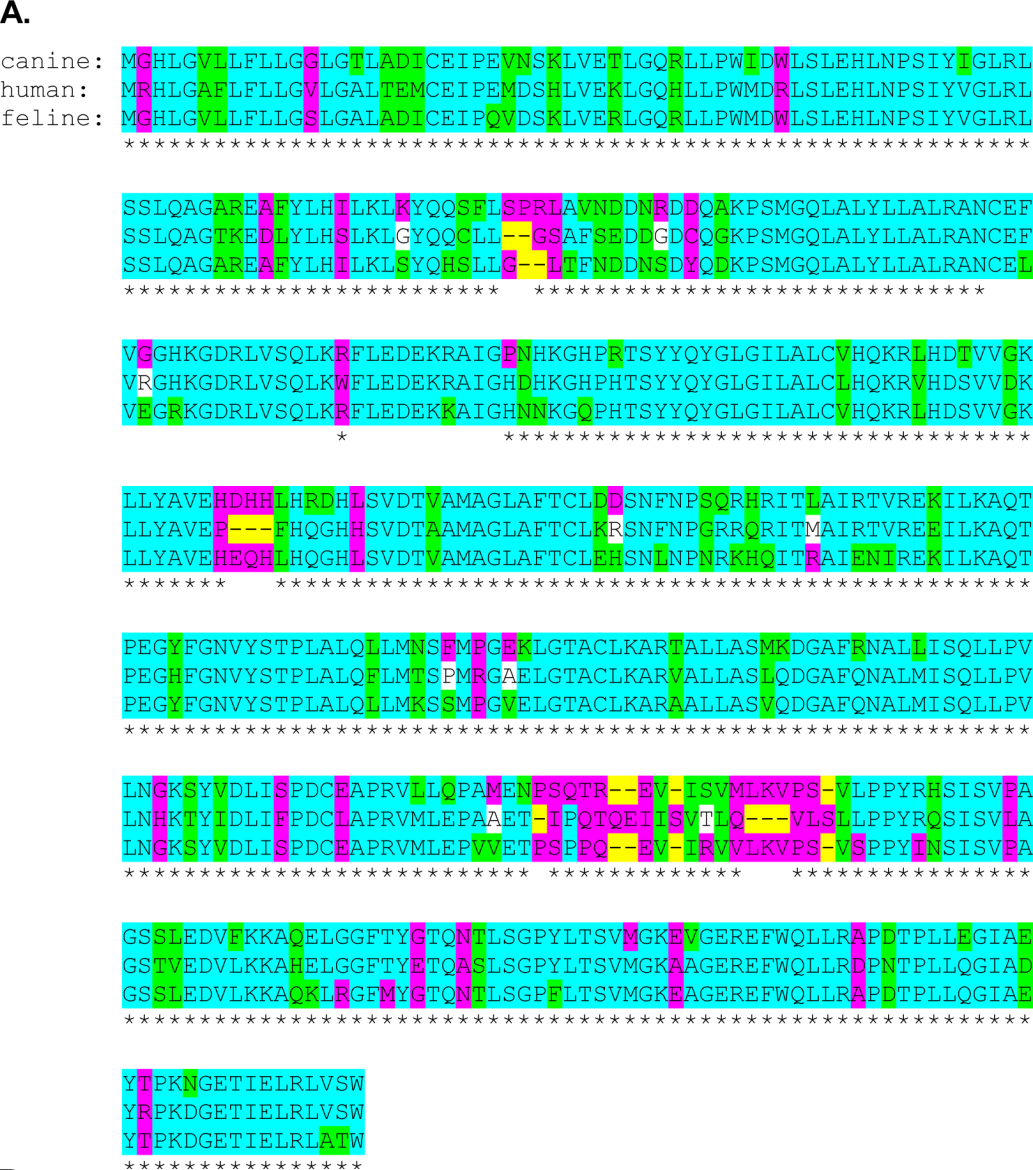

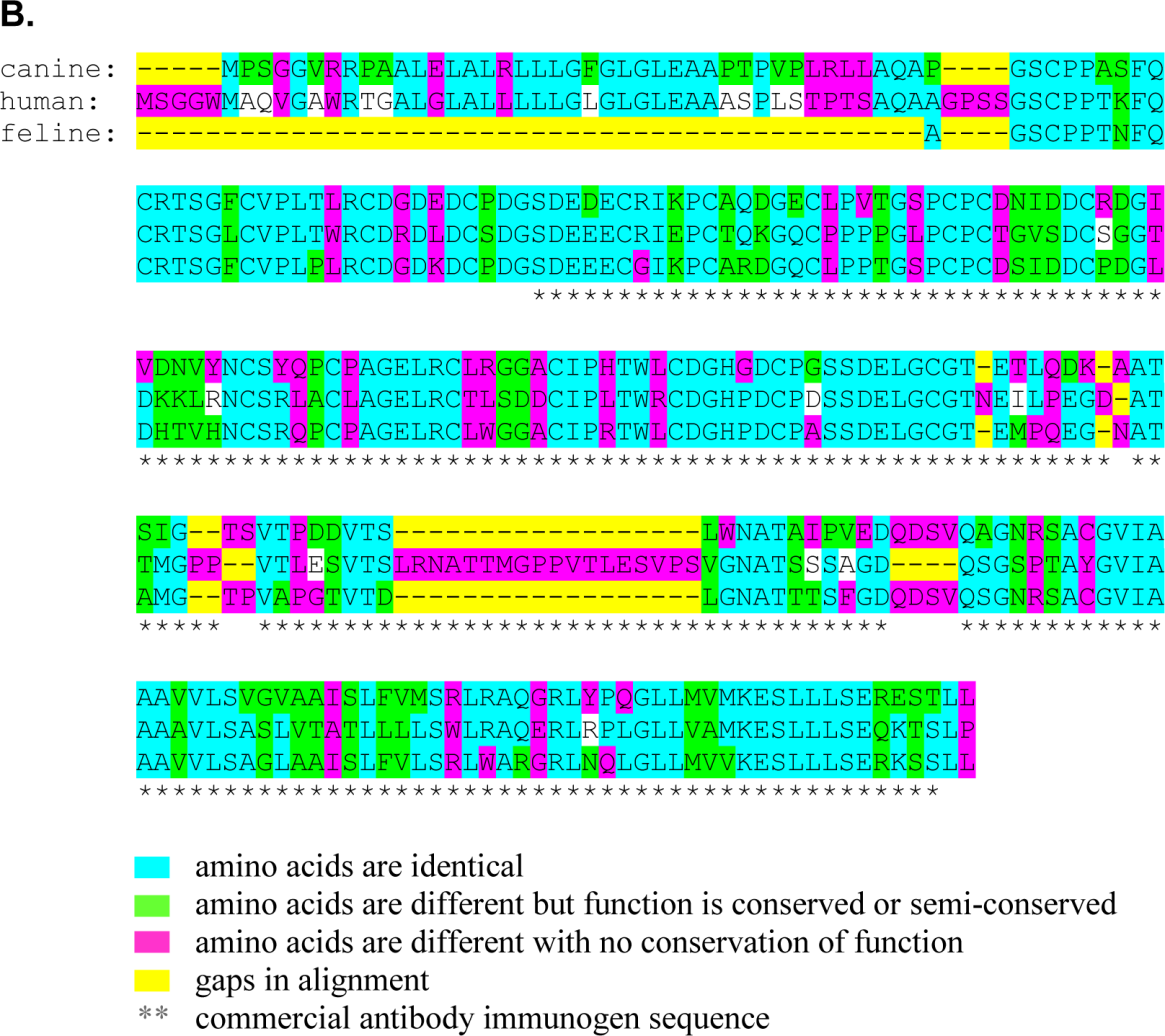
Conservation of TCII and TCII-R amino acid sequence homology between human and canine and between human and feline species Sequences are illustrated using the 2014 Ensembl Comparative Genomics Orthologue alignments. (A) TCII amino acid sequence. (B) TCII-R amino acid sequence.

The primary TCII and TCII-R antibodies used in this study were commercially available rabbit polyclonal antibodies raised to human TCII and TCII-R antigens, and this study is the first to evaluate them in canine and feline tissues. Both TCII and TCII-R proteins are expressed in all mammals. Based on comparison of primary sequences, the amino acids involved in TCII-Cbl binding, the cysteine residues involved in disulfide bonds, the TCII-R-encoding gene structure and the TCII-R flanking DNA, all appear to be highly conserved across species [13]. We evaluated human, canine and feline protein sequence files from the Ensembl genome browser (Ensembl 2014, release 76, URL http://useast.ensembl.org/index.html) using the Ensembl Comparative Genomics Orthologue function to identify regions of similar protein alignment between the biologic sequences for the TCII and TCII-R genes (Figure 5). We identified 73% sequence similarity between human TCII and both canine/feline TCII, and 83% sequence similarity between canine and feline TCII. There was a 57% (canine) and 62% (feline) TCII-R protein sequence similarity to human, and a 76% similarity between canine and feline TCII-R sequences. The high degree of similarity between human, canine and feline TCII and TCII-R protein sequences, as well as length of similar antigen peptide regions recognized by the TCII and TCII-R primary antibodies permitted successful binding of human-specific primary antibodies in the canine and feline tissues used for IHC staining in this study.

The primary Ki-67 antibody used in this study was a commonly used, commercially available mouse monoclonal antibody raised to the human Ki-67 antigen. This antibody has been used extensively to evaluate the IHC expression of Ki-67 in proliferating cells in a number of species, including human [33-35], canine [31, 36, 37] and feline [38-40]. Although the Ensembl genome browser identifies only 50% similarity between human and both canine/feline Ki-67 protein sequences, measurement of Ki-67 using a human-specific primary antibody in canine and feline tumor tissues has been widely used to characterize tumor histologic and molecular features [30, 31, 41, 42], establish tumor grade [28], aid in treatment selection [41] and determine prognosis [31, 40, 41, 43], as well as to evaluate the effect of proliferation on expression of a number of relatively common tumor biomarkers such as estrogen receptor [32, 44], progesterone receptor [32, 44], human epidermal growth factor receptor (HER2) [29, 32], receptor tyrosine kinase (cKit) [45, 46] and vascular endothelial growth factor (VEGF) [47]. Unfortunately, lack of standardization in processing, staining, imaging and analysis techniques across all of these studies precludes the direct comparison of previously calculated canine and feline Ki-67 values to the Ki-67 values quantitated in this study.

Every malignant tumor tissue evaluated in this study stained positively for TCII and TCII-R, with the exception of one canine osteosarcoma case that did not demonstrate staining for TCII, and one feline intestinal mast cell tumor case that did not demonstrate staining for TCII-R. Low sample cellularity may have been a contributing factor for inapparent staining in these two cases. Measurable levels of TCII and TCII-R identified in all other malignant tumor tissues suggests an intracellular demand for Cbl by each canine and feline tumor evaluated. Cobalamin largely exerts its effects on cell, and ultimately tumor, growth by acting as a cofactor in methionine synthase-mediated methylation of homocysteine to methionine. The majority of methionine is converted to S-adenosylmethionine, which serves as a universal methyl donor for DNA, RNA, phospholipids and metabolites [23]. The Cbl-dependent methionine synthase reaction also results in regeneration of tetrahydrofolate, which is required for synthesis of the thymidine and purine/pyrimidine constituents of DNA [48]. Quantifiable expression of the Cbl transport protein and cell surface receptor in every canine and feline malignant tumor that we evaluated suggests a universal, obligate role of Cbl to support DNA synthesis for tumor proliferative growth.

The most striking finding in this study was the highly significant increase in TCII, TCII-R and Ki-67 expression in malignant tumor tissues compared to normal tissues located within millimeters from the tumor itself. These results clearly demonstrate specific up-regulation of TCII and TCII-R expression by proliferating malignant tissues across multiple tumor types in both canine and feline species, and provide compelling support for use of TCII and TCII-R proteins as cancer biomarkers, as well as the use of Cbl-bound diagnostic and therapeutic agents in clinical cancer medicine. The results of this study agree with results of previous research studies that have demonstrated increased TCII [21, 49, 50] and TCII-R [18, 22, 23] generation by malignant tissues.

Although background uptake of Cbl-bound diagnostic and therapeutic bioconjugates by healthy tissues has been considered a concern with their clinical use [51], the demonstrated over-expression of TCII-R in malignant tissues compared to immediately adjacent normal tissues suggests a disproportionate channeling of Cbl-bound agents to tumor cells, thereby limiting uptake by healthy tissues. In a case series of dogs with naturally occurring tumors, treatment with a cobalamin-based anti-tumor agent over periods ranging from 5 months to 3 years (on long term follow up) resulted in tumor remission in all dogs with no discernible adverse effects, suggesting that uptake of the Cbl-based drug by healthy tissues was minimal [52]. While diversion of Cbl to tumor tissues is favorable for safety of Cbl-bound agents, it does bring to light the dilemma of addressing low serum Cbl levels, which has been identified in human [53], canine [54] and feline [55] patients with cancer. In these patients, it is possible that the majority of supplementally-administered Cbl will be forfeited to tumor cell proliferation rather than used to support normal systemic metabolic processes. Thus, successful delivery of supplemental Cbl in hypocobalaminemic patients will depend on an uptake mechanism that is not up-regulated by tumor cells, and to our knowledge, such a mechanism has yet to be defined. Immunohistochemical measurement of tumor TCII-R expression may be valuable in determining the practicality of Cbl supplementation in hypocobalaminemic cancer patients.

Expression of TCII tended to be higher than that of TCII-R in both canine and feline malignant tumor tissues as well as in adjacent normal tissues. Conversely, in 34 human malignant xenograft tissues that were identically processed, stained and analyzed, we found that expression of TCII-R was greater than that of TCII in over half of tumor tissues [24]. In humans, approximately 75% of Cbl in the circulation is bound to a protein known as transcobalamin I (TCI; haptocorrin), and only 25% is bound to TCII [56]; it is the TCII-bound fraction of Cbl that is available for cellular uptake and use in methionine synthase reactions. Interestingly, early research evaluating Cbl-binding proteins in dogs and cats suggested that the majority of circulating Cbl in these species is bound to TCII rather than to TCI [57, 58]. It is possible that in the canine and feline tissues of this reported study, TCII expression was observed to be greater than TCII-R expression simply because of the comparatively high levels of TCII present. Additionally, use of rabbit anti-human polyclonal antibodies with higher canine/feline sequence similarity for TCII compared to TCII-R may have resulted in greater antibody binding and increased chromagen expression for TCII, resulting in higher calculated staining values for TCII. Lastly, use of naturally occurring tissues in this study rather than xenograft tissues as used in the human study may have contributed to this observed difference. Additional work to assess the presence and contribution of the Cbl-binding proteins in dogs and cats and the development of species-specific antibodies to TCII and TCII-R is warranted to further explore comparative Cbl transport and delivery mechanisms in non-human species.

In both canine and feline malignant tumor tissues, a significant and near-identical correlation was identified between TCII and TCII-R staining values, demonstrating that increases in tumor tissue TCII expression were matched by increases in TCII-R expression. A period of approximately 4 months separated the staining and analysis of canine and feline tumor tissues, and the finding of similar TCII/TCII-R correlation coefficients in both canine and feline samples suggests that the standardized protocol used was repeatable and enabled comparison of TCII and TCII-R staining values both within and between species. A strong correlation between TCII and TCII-R protein expression was expected, as up-regulation of both TCII and TCII-R is necessary to facilitate the delivery and absorption of Cbl, which is highly coveted by proliferating cells. The TCII and TCII-R proteins are critically dependent upon one another for cellular uptake of Cbl, and endocytosis of large quantities of Cbl required by actively dividing tumor cells is not possible if either of these proteins is absent or is present in substantially reduced amounts. An intimate coordination between the mechanisms responsible for up-regulation of TCII and TCII-R expression in malignant tumor tissues are supported by results of this study. However, the specific biologic processes which facilitate synchronous up-regulation of these two proteins have yet to be deciphered.

All malignant tumor tissues in this study exhibited positive nuclear staining for Ki-67, indicating active cellular proliferation at the time of tumor resection or biopsy. As with TCII and TCII-R, Ki-67 expression was significantly higher in malignant tumor tissues compared to immediately adjacent normal tissues in both canine and feline species. These results are consistent with results of previously published studies in dogs and cats that have demonstrated higher Ki-67 staining in a variety of malignant tissues compared to benign tumors, hyperplastic/dysplastic tissues and normal tissues [27, 28, 30, 31, 59]. We identified a moderate correlation between Ki-67 and TCII expression in canine malignant tumor tissues, and between Ki-67 and TCII-R in both canine and feline malignant tumor tissues. Lack of a more substantial correlation between Ki-67 and TCII/TCII-R expression in malignant tumor tissues of this study was interesting, since cellular uptake of Cbl is vital for DNA synthesis associated with rapid cellular division and proliferation. However, similar results have been found in other canine and feline studies evaluating the relationship between Ki-67 and various tumor biomarkers that would be anticipated to be up-regulated in proliferative tissues [29, 40, 47].

**Fig.6:**
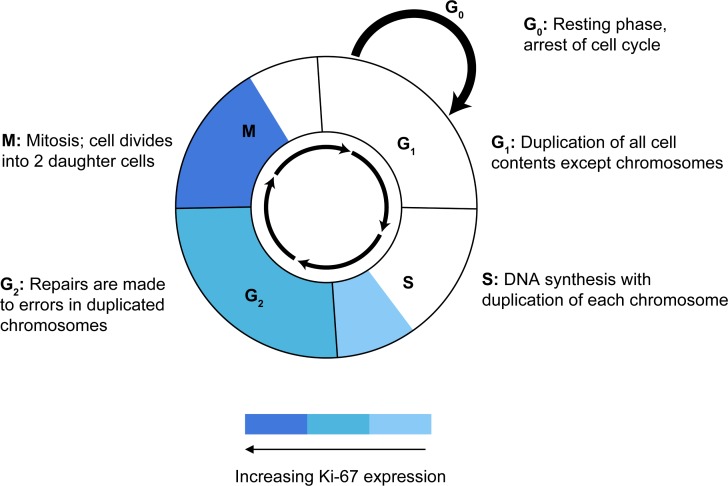
Ki-67 expression during phases of the cell cycle An increase in Ki-67 expression is noted during the late S and G_2_ phases of the cell cycle, with expression peaking during the M phase.

There are several possible factors that may affect correlation between Ki-67 and TCII /TCII-R expression. Ki-67 is a nuclear protein that is expressed to some degree during all active phases of the cell cycle (late G_1_, S, G_2_ and M phases) but is absent from resting cells (G_0_) [60]. Although little is known about its specific function, Ki-67 protein expression is believed to be an absolute requirement for progression through the cell division cycle [61]. Expression of Ki-67 is tightly regulated, and has been shown to increase during the latter half of the S and G_2_ phases and peak in the M phase of the cell cycle [60, 62] (Figure 6). In contrast, while it has been shown that TCII-R expression is highest during the active growth phase in proliferating cells [63], very little is known about the timing of peak TCII or TCII-R expression during specific phases of the cell cycle. It is possible that TCII and TCII-R expression may be highest in the early phases of the cell cycle (G_1_, early S) in order to ensure adequate levels of intracellular Cbl necessary to support upcoming DNA replication. Asynchrony between cell cycle phases of peak Ki-67 and TCII/TCII-R activity may affect the ability to identify a stronger correlation between expression of these proteins. Additionally, it has been suggested that because late G_1_ and very early S phase cells express Ki-67 antigen levels at just slightly higher than background levels, some late G_1_ cells may be erroneously classified as non-cycling cells [62], which could artificially decrease calculated Ki-67 staining values and affect the interpretation of its correlation with expression of other tumor proteins. Furthermore, the 60-90 minute half lives of Ki-67 and TCII are relatively short [60, 62, 64] in comparison to the 8 h half life of TCII-R [65], thereby affecting the ability to uniformly capture timing of peak expression for each protein. In addition, it has been suggested that cellular proliferation may be more accurately measured by taking into consideration both number of cycling cells present (“growth fraction”, demonstrated by IHC staining for Ki-67), as well as rate of progression through the cell cycle (“generation time”, which can be demonstrated using IHC silver stains for DNA segments encoding ribosomal RNA, known as argyrophilic nucleolar organizing region proteins; AgNOR) [45, 59]. Since growth fraction (the number of cycling cells) and generation time (the speed at which cells are cycling) are largely independent of one another, measurement of both Ki-67 and AgNOR proteins may be necessary to more accurately determine the proliferative status of a tumor [45] and to help identify a correlation with other proliferation-dependent tumor proteins. Lastly, intratumoral heterogeneity has been shown to influence distribution of Ki-67 expression [66]. As a malignant tumor acquires progressive changes through clonal evolution, some areas of the tumor may demonstrate a certain level of protein biomarker expression while other areas of the same tumor may express that same biomarker to a greater or lesser degree [67]. In the study reported here, a separate slide was used to stain and evaluate expression of each protein biomarker, and measurement of expression was calculated in a representative area of malignant tumor tissue with highest, consistent staining. Based on these staining and imaging criteria, measurement of expression of each protein biomarker was obtained at slightly different regions of tumor tissue, and attempts to identify correlation between these proteins may have been affected by local tissue heterogeneity. Simultaneous visualization of multiple protein biomarkers on a single tissue section site is now possible through automated multiplex IHC staining and multispectral imaging procedures [68]. Use of these procedures may provide a means of capturing the interaction of tumor biomarkers at a single site and make possible a more reliable measure of their correlation.

The variation observed in TCII, TCII-R and Ki-67 expression both within and between malignant tumor types was not unexpected since genotypic, phenotypic and environmental influences provide each tumor with unique biologic characteristics. Intra- and inter-tumoral variation in protein biomarker expression has been identified in other studies [28, 42, 69], and affords strong support for the current trend towards personalized cancer medicine.

Three similar malignant tumor types were evaluated in both canine and feline species. With the specific exception of feline vaccine-associated fibrosarcomas, there was no significant difference in TCII, TCII-R and Ki-67 staining values between canine and feline species for any of the three similar tumor types. Among similar tumor types, distinctive trends in protein biomarker expression were observed: TCII, TCII-R and Ki-67 staining values for canine and feline urinary bladder transitional cell carcinomas were consistently high, while staining values for canine and feline soft tissue sarcomas were routinely low. This finding suggests that malignant tumor types may possess characteristic metabolic profiles, and these profiles, in conjunction with immunohistochemical quantification of TCII, TCII-R and Ki-67 expression, may play an important role in establishing inclusion criteria for clinical trials evaluating Cbl-based anti-tumor agents and ultimately in selection of optimal treatment regimens with either single agent or combination therapies.

Observation of average and median TCII, TCII-R and Ki-67 staining values between species demonstrated generally higher values in feline malignant tumor tissues than in canine malignant tumor tissues. These IHC findings suggest that feline malignancies can be particularly vigorous and aggressive, an effect that is often observed clinically, and they support a potential association between expression of these biomarkers and clinical tumor behavior. Use of the TCII, TCII-R and Ki-67 protein biomarkers to confirm the presence of malignant tumor tissue and to expediently guide treatment selection may be vital in helping to improve outcomes for feline cancer patients.

In summary, the results of this study have quantitatively established a significant, synchronous up-regulation of the Cbl transport protein and cell surface receptor in naturally occurring, proliferating malignant tumor tissues. The study data has also suggested that expression of these protein biomarkers can vary both within and between tumor types, and supports the increasing necessity of personalized cancer care. Additional studies will be important to i) validate up-regulation of these proteins in naturally occurring human malignant tumor tissues; ii) to verify the use of human patient peri-tumoral normal tissues as individual controls; iii) to determine the applicability of quantifying these biomarkers from easily obtained needle aspirates or cytologic smears; iv) to evaluate the effects of chemotherapeutic and radiation treatment on biomarker expression, and v) to assess the correlation between biomarker expression and clinical outcome. Standardized IHC quantification of TCII, TCII-R and Ki-67 may become clinically useful to assist in the selection of patients for treatment with Cbl-based anti-tumor therapies in the clinical trial setting, and to aid in the diagnosis, treatment and monitoring of individual human and companion animal tumors.

## METHODS

### Tissue samples

Patient-derived tissue samples were obtained from archived malignant tumor tissues obtained at the time of biopsy or surgical resection (VDx Veterinary Pathology Services, Davis, CA, USA). Three cases each of ten canine malignant tumor types (n = 30) and twelve feline malignant tumor types (n = 36) were selected based on previously established histopathologic diagnosis. Formalin-fixed, paraffin-embedded tissues were sectioned at 3 microns, mounted on positively charged glass slides and dried. One slide from each tumor was stained with hematoxylin and eosin and examined by a board-certified pathologist to confirm the histopathologic diagnosis and to verify that cut location included representative tumor tissue as well as adjacent normal, non-malignant tissue.

### Immunohistochemical staining procedure for TCII and TCII-R

Antibody dilutions were determined by using incremental dilutions and comparing results to those obtained for quantifying these proteins in previously published studies [22, 24]. Slides were deparaffinized and endogenous peroxidase activity was quenched by incubation of the slides in 3% hydrogen peroxide. Slides were then placed in citrate buffer of pH 6.0 and heat-induced epitope retrieval was accomplished using a Dako Pascal pressure chamber (Dako North America Inc., Carpinteria, CA, USA). Once cooled, slides were placed in SuperSensitive ^TM^ Wash Buffer 20X (BioGenex, Fremont, CA, USA) and loaded onto a Dako Autostainer Plus (Dako North America Inc.). Slides were incubated for 1 hour at room temperature with one of the following primary antibodies: rabbit polyclonal antibody to TCII (TCN2; GenBank accession number BC011239; 1:50 dilution canine; 1:25 dilution feline; Proteintech Group, Chicago, IL, USA) or with rabbit polyclonal antibody to TCII-R (CD320; GenBank accession number BC000668; 1:50 dilution canine; 1:25 dilution feline; Proteintech Group, Chicago) using Common Antibody diluent (BioGenex). Slides were then incubated for 1 hour with PromARK^TM^ Rabbit-on-Canine horseradish peroxidase (HRP) polymer secondary antibody (Biocare Medical, Concord, CA, USA). Immunostaining visualization was achieved by immersion of slides in diaminobenzidine (DAB) (Biocare Medical) and counterstaining with Mayer's hematoxylin. Slides were dehydrated and cleared, then mounted with Permount^TM^ synthetic mounting medium (Fisher Scientific, Waltham, MA, USA). To ensure the specificity of immunostaining, negative control slides were prepared by omitting the primary antibody.

### Immunohistochemical staining procedure for Ki-67

Slides were deparaffinized, treated with 0.3% hydrogen peroxide in methanol and rehydrated. Slides were then placed into a citrate buffer antigen-retrieval solution (Dako North America Inc.). Heat-induced epitope retrieval was accomplished using a steamer (Black and Decker, New Britain, CT, USA). Slides were blocked with 10% normal horse serum (Quad Five, Ryegate, MT, USA) in phosphate buffered saline with 0.02% Tween 20 (Sigma Aldrich, St. Louis, MO, USA ). For each tumor, one slide was incubated for 1 hour with a mouse monoclonal primary antibody to Ki-67 (Clone MIB-1; 1:40 dilution canine and feline; Dako North America, Inc.); a second slide was not exposed to the primary antibody in order to serve as a negative control. Slides were next incubated with a horse anti-mouse secondary antibody (Biocare Medical), followed by incubation with streptavidin-horse radish peroxidase (Biocare Medical). Slides were then developed with Nova Red (Vector Labs, Burlingame, CA, USA), counterstained in Mayers hematoxylin and air dried.

### Microscopic evaluation and imaging

Slides were examined by light microscopy using an Olympus BH-2 microscope (Olympus, Center Valley, PA, USA) fitted with a 12.5 megapixel, 12-bit digital color camera with Peltier cooling and DP Manager and Controller software (Olympus, version 3.0). An ultra-high resolution mode (4080 × 3072) was used, and light settings were standardized for all imaging sessions. TCII and TCII-R positive cells exhibited DAB-positive (brown) staining, and Ki-67 positive cells exhibited Nova Red-positive (red-brown) staining; negative cells stained with hematoxylin counterstain only. Stained slides were reviewed by a board-certified pathologist. A representative area of solid tumor devoid of connective tissue and ischemic necrosis and with even distribution of cells was selected and digitally photographed for each tumor tissue sample. Similarly, a representative, uniformly-stained area of adjacent non-malignant tissue was also photographed for each sample. Images for TCII and TCII-R analysis were obtained at 10x magnification (200 micron scale bar), and images for Ki-67 analysis were obtained at 40x magnification (50 micron scale bar). JPEG format was used to capture all digital images.

### Digital image analysis for TCII and TCII-R

For each image, the color deconvolution method was used to isolate TCII and TCII-R positive DAB-stained cells from TCII and TCII-R negative hematoxylin-stained cells. DAB and hematoxylin were digitally separated using ImageJ software (version 1.46c; WS Rasband, National Institutes of Health, Bethesda, MD, USA, URL http://rsb.info.nih.gov/ij/) and an ImageJ plugin for color deconvolution [70], which calculated the contribution of DAB and hematoxylin based on stain-specific red-green-blue (RGB) absorption [71]. Following deconvolution, scale was set to the 200 micron scale bar on each image. The deconvoluted image was subjected to histogram analysis, with lower threshold set at 10 and upper threshold set at 100. For each image, three fields of consistent staining were selected and measured using 200 × 200 pixel boxes. A value was assigned to each field using ImageJ software, and the average value of all three fields was used to assign a staining value (arbitrary units) to each image.

### Digital image analysis for Ki-67

Staining was evaluated using ImageJ and the ImmunoRatio plugin [72] optimized for Ki-67 nuclear staining and utilizing the color deconvolution method described above [71]. Background correction was performed using an image from an empty slide background (blank field image) to address image color balance and uneven illumination. The number of Ki-67 positive NovaRed-stained cells over the total number of cells was calculated and used to assign a staining value (%) to each image.

### Statistical analysis

The Spearman rank correlation co-efficient was used as a non-parametric measure of statistical dependence between TCII, TCII-R and Ki-67 expression (Free Statistics Software; Office for Research Development and Education, version 1.1.23-r7, URL http://www.wessa.net). An upaired two-tailed Student's t-test with a pooled estimator of variance was used to determine statistical difference in the expression of TCII, TCII-R and Ki-67 between i) malignant and normal tissues, and ii) canine and feline tissues (Sigma Plot 10.0; SPSS, Chicago, IL, USA). Significance was set at p < 0.05.

## SUPPLEMENTARY MATERIAL AND DATA



## References

[1] McCormick T MK, Hehenberger M (2007). The evolving role of biomarkers: Focusing on patients from research to clinical practice. Biomarker Summit III.

[2] Sarker D, Pacey S, Workman P (2007). Use of pharmacokinetic/pharmacodynamic biomarkers to support rational cancer drug development. Biomark Med.

[3] Biomarkers Definitions Working Group (2001). Biomarkers and surrogate endpoints: Preferred definitions and conceptual framework. Clin Pharmacol Ther.

[4] Khleif SN, Doroshow JH, Hait WN (2010). AACR-FDA-NCI Cancer Biomarkers Collaborative consensus report: Advancing the use of biomarkers in cancer drug development. Clin Cancer Res.

[5] Dunstan RW, Wharton KA, Quigley C, Lowe A (2011). The use of immunohistochemistry for biomarker assessment--can it compete with other technologies?. Toxicol Pathol.

[6] Makawita S, Diamandis EP (2010). The bottleneck in the cancer biomarker pipeline and protein quantification through mass spectrometry-based approaches: current strategies for candidate verification. Clin Chem.

[7] Rhea JM, Molinaro RJ (2011). Cancer biomarkers: surviving the journey from bench to bedside. Med Lab Obs.

[8] Fuzery AK, Levin J, Chan MM, Chan DW (2013). Translation of proteomic biomarkers into FDA approved cancer diagnostics: issues and challenges. Clin Proteomics.

[9] Seetharam B, Li N (2000). Transcobalamin II and its cell surface receptor. Vitam Horm.

[10] Quadros EV, Rothenberg SP, McLoughlin P (1996). Characterization of monoclonal antibodies to epitopes of human transcobalamin II. Biochem Biophys Res Commun.

[11] Scott JM, Weir DG (1998). Folic acid, homocysteine and one-carbon metabolism: a review of the essential biochemistry. J Cardiovasc Risk.

[12] Russell-Jones G, McTavish K, McEwan J, Rice J, Nowotnik D (2004). Vitamin-mediated targeting as a potential mechanism to increase drug uptake by tumours. J Inorg Biochem.

[13] Quadros EV, Sequeira JM (2013). Cellular uptake of cobalamin: transcobalamin and the TCblR/CD320 receptor. Biochimie.

[14] Frater-Schroder M, Porck HJ, Erten J, Muller MR, Steinmann B, Kierat L, Arwert F (1985). Synthesis and secretion of the human vitamin B12-binding protein, transcobalamin II, by cultured skin fibroblasts and by bone marrow cells. Biochim Biophys Acta.

[15] Quadros EV, Rothenberg SP, Jaffe EA (1989). Endothelial cells from human umbilical vein secrete functional transcobalamin II. Am J Physiol.

[16] Rothenberg SP, Quadros EV (1995). Transcobalamin II and the membrane receptor for the transcobalamin II-cobalamin complex. Baillieres Clin Haematol.

[17] Lindemans J, Kroes AC, van Geel J, van Kapel J, Schoester M, Abels J (1989). Uptake of transcobalamin II-bound cobalamin by HL-60 cells: effects of differentiation induction. Exp Cell Res.

[18] Amagasaki T, Green R, Jacobsen DW (1990). Expression of transcobalamin II receptors by human leukemia K562 and HL-60 cells. Blood.

[19] Collins DA, Hogenkamp HP, O'Connor MK, Naylor S, Benson LM, Hardyman TJ, Thorson LM (2000). Biodistribution of radiolabeled adenosylcobalamin in patients diagnosed with various malignancies. Mayo Clin Proc.

[20] Rabinowitz R, Rachmilewitz B, Rachmilewitz M, Schlesinger M (1982). Production of transcobalamin II by various murine and human cells in culture. Isr J Med Sci.

[21] Hall CA, Green-Colligan PD, Begley JA (1985). Synthesis of transcobalamin II by cultured human hepatocytes. Biochim Biophys Acta.

[22] Bauer JA, Morrison BH, Grane RW, Jacobs BS, Dabney S, Gamero AM, Carnevale KA, Smith DJ, Drazba J, Seetharam B, Lindner DJ (2002). Effects of interferon beta on transcobalamin II-receptor expression and antitumor activity of nitrosylcobalamin. J Natl Cancer Inst.

[23] Jiang W, Sequeira JM, Nakayama Y, Lai SC, Quadros EV (2010). Characterization of the promoter region of TCblR/CD320 gene, the receptor for cellular uptake of transcobalamin-bound cobalamin. Gene.

[24] Sysel AM, Valli VE, Nagle RB, Bauer JA (2013). Immunohistochemical quantification of the vitamin B12 transport protein (TCII), cell surface receptor (TCII-R) and Ki-67 in human tumor xenografts. Anticancer Res.

[25] Rickes EL, Brink NG, Koniuszy FR, Wood TR, Folkers K (1948). Crystalline Vitamin B12. Science.

[26] Scott JM, Molloy AM (2012). The discovery of vitamin B(12). Ann Nutr Metab.

[27] Pena LL, Nieto AI, Perez-Alenza D, Cuesta P, Castano M (1998). Immunohistochemical detection of Ki-67 and PCNA in canine mammary tumors: relationship to clinical and pathologic variables. J Vet Diag Invest.

[28] Dias Pereira P, Carvalheira J, Gartner F (2004). Cell proliferation in feline normal, hyperplastic and neoplastic mammary tissue--an immunohistochemical study. Vet J.

[29] Rasotto R, Caliari D, Castagnaro M, Zanetti R, Zappulli V (2011). An Immunohistochemical study of HER-2 expression in feline mammary tumours. J Comp Pathol.

[30] Millanta F, Lazzeri G, Mazzei M, Vannozzi I, Poli A (2002). MIB-1 labeling index in feline dysplastic and neoplastic mammary lesions and its relationship with postsurgical prognosis. Vet Pathol.

[31] Pereira RS, Schweigert A, Dias de Melo G, Fernandes FV, Sueiro FA, Machado GF (2013). Ki-67 labeling in canine perianal glands neoplasms: a novel approach for immunohistological diagnostic and prognostic. BMC Vet Res.

[32] Burrai GP, Mohammed SI, Miller MA, Marras V, Pirino S, Addis MF, Uzzau S, Antuofermo E (2010). Spontaneous feline mammary intraepithelial lesions as a model for human estrogen receptor- and progesterone receptor-negative breast lesions. BMC Cancer.

[33] Dowsett M, Nielsen TO, A'Hern R, Bartlett J, Coombes RC, Cuzick J, Ellis M, Henry NL, Hugh JC, Lively T, McShane L, Paik S, Penault-Llorca F (2011). Assessment of Ki67 in breast cancer: recommendations from the International Ki67 in Breast Cancer working group. J Natl Cancer Inst.

[34] Naz E, Mirza T, Aziz S, Ali A, Danish F (2011). Correlation of Ki 67 proliferative index with clinical and pathological features on tissue sections of non Hodgkins lymphoma by immunostaining. J Pak Med Assoc.

[35] Brandao RD, Veeck J, Van de Vijver KK, Lindsey P, de Vries B, van Elssen CH, Blok MJ, Keymeulen K, Ayoubi T, Smeets HJ, Tjan-Heijnen VC, Hupperets PS (2013). A randomised controlled phase II trial of pre-operative celecoxib treatment reveals anti-tumour transcriptional response in primary breast cancer. Breast Cancer Res.

[36] Bergin IL, Smedley RC, Esplin DG, Spangler WL, Kiupel M (2011). Prognostic evaluation of Ki67 threshold value in canine oral melanoma. Vet Pathol.

[37] Santos AA, Lopes CC, Ribeiro JR, Martins LR, Santos JC, Amorim IF, Gartner F, Matos AJ (2013). Identification of prognostic factors in canine mammary malignant tumours: a multivariable survival study. BMC Vet Res.

[38] Morris JS, Nixon C, Bruck A, Nasir L, Morgan IM, Philbey AW (2008). Immunohistochemical expression of TopBP1 in feline mammary neoplasia in relation to histological grade, Ki67, ERalpha and p53. Vet J.

[39] Sakai H, Mori T, Iida T, Tokuma Y, Maruo K, Masegi T (2008). Immunohistochemical features of proliferative marker and basement membrane components of two feline inductive odontogenic tumours. J Feline Med Surg.

[40] Bergkvist GT, Argyle DJ, Morrison L, MacIntyre N, Hayes A, Yool DA (2011). Expression of epidermal growth factor receptor (EGFR) and Ki67 in feline oral squamous cell carcinomas (FOSCC). Vet Comp Oncol.

[41] Melzer K, Guscetti F, Rohrer Bley C, Sumova A, Roos M, Kaser-Hotz B (2006). Ki67 reactivity in nasal and periocular squamous cell carcinomas in cats treated with electron beam radiation therapy. J Vet Intern Med.

[42] Millanta F, Fratini F, Corazza M, Castagnaro M, Zappulli V, Poli A (2002). Proliferation activity in oral and cutaneous canine melanocytic tumours: correlation with histological parameters, location, and clinical behaviour. Res Vet Sci.

[43] Buishand FO, Kik M, Kirpensteijn J (2010). Evaluation of clinico-pathological criteria and the Ki67 index as prognostic indicators in canine insulinoma. Vet J.

[44] Mandara MT, Ricci G, Rinaldi L, Sarli G, Vitellozzi G (2002). Immunohistochemical identification and image analysis quantification of oestrogen and progesterone receptors in canine and feline meningioma. J Comp Pathol.

[45] Webster JD, Yuzbasiyan-Gurkan V, Miller RA, Kaneene JB, Kiupel M (2007). Cellular proliferation in canine cutaneous mast cell tumors: associations with c-KIT and its role in prognostication. Vet Pathol.

[46] Sabattini S, Bettini G (2010). Prognostic value of histologic and immunohistochemical features in feline cutaneous mast cell tumors. Vet Pathol.

[47] Matiasek LA, Platt SR, Adams V, Scase TJ, Keys D, Miller J, Adamo F, Long S, Matiasek K (2009). Ki-67 and vascular endothelial growth factor expression in intracranial meningiomas in dogs. J Vet Intern Med.

[48] Bailey LB, Gregory JF (1999). Folate metabolism and requirements. J Nutr.

[49] Rachmilewitz B, Sulkes A, Rachmilewitz M, Fuks Z (1981). Serum transcobalamin II levels in breast carcinoma patients. Isr J Med Sci.

[50] Jensen HS, Gimsing P, Pedersen F, Hippe E (1983). Transcobalamin II as an indicator of activity in metastatic renal adenocarcinoma. Cancer.

[51] Clardy SM, Allis DG, Fairchild TJ, Doyle RP (2011). Vitamin B12 in drug delivery: breaking through the barriers to a B12 bioconjugate pharmaceutical. Expert Opin Drug Deliv.

[52] Bauer JA, Frye G, Bahr A, Gieg J, Brofman P (2010). Anti-tumor effects of nitrosylcobalamin against spontaneous tumors in dogs. Invest New Drugs.

[53] Tong SY, Kim MK, Lee JK, Lee JM, Choi SW, Friso S, Song ES, Lee KB, Lee JP (2011). Common polymorphisms in methylenetetrahydrofolate reductase gene are associated with risks of cervical intraepithelial neoplasia and cervical cancer in women with low serum folate and vitamin B12. Cancer Cause Control.

[54] Cook AK, Wright ZM, Suchodolski JS, Brown MR, Steiner JM (2009). Prevalence and prognostic impact of hypocobalaminemia in dogs with lymphoma. J Am Vet Med Assoc.

[55] Kook PH, Lutz S, Sewell AC, Bigler B, Reusch CE (2012). [Evaluation of serum cobalamin concentration in cats with clinical signs of gastrointestinal disease]. Schweiz Arch Tierh.

[56] Hall CA (1975). Transcobalamins I and II as natural transport proteins of vitamin B12. J Clin Invest.

[57] Rappazzo ME, Hall CA (1972). Cyanocobalamin transport proteins in canine plasma. Am J Physiol.

[58] Linnell JC, Collings L, Down MC, England JM (1979). Distribution of endogenous cobalamin between the transcobalamins in various mammals. Clin Sci (Lond).

[59] Bauer NB, Zervos D, Moritz A (2007). Argyrophilic nucleolar organizing regions and Ki67 equally reflect proliferation in fine needle aspirates of normal, hyperplastic, inflamed, and neoplastic canine lymph nodes (n = 101). J Vet Intern Med.

[60] Scholzen T, Gerdes J (2000). The Ki-67 protein: from the known and the unknown. J Cell Physiol.

[61] Schluter C, Duchrow M, Wohlenberg C, Becker MH, Key G, Flad HD, Gerdes J (1993). The cell proliferation-associated antigen of antibody Ki-67: a very large, ubiquitous nuclear protein with numerous repeated elements, representing a new kind of cell cycle-maintaining proteins. J Cell Biol.

[62] Bruno S, Darzynkiewicz Z (1992). Cell cycle dependent expression and stability of the nuclear protein detected by Ki-67 antibody in HL-60 cells. Cell Prolif.

[63] Hall CA, Colligan PD, Begley JA (1987). Cyclic activity of the receptors of cobalamin bound to transcobalamin II. J Cell Physiol.

[64] Schneider RJ, Burger RL, Mehlman CS, Allen RH (1976). The role and fate of rabbit and human transcobalamin II in the plasma transport of vitamin B12 in the rabbit. J Clin Invest.

[65] Youngdahl-Turner P, Mellman IS, Allen RH, Rosenberg LE (1979). Protein mediated vitamin uptake. Adsorptive endocytosis of the transcobalamin II-cobalamin complex by cultured human fibroblasts. Exp Cell Res.

[66] Jakobsen JN, Santoni-Rugiu E, Ravn J, Sorensen JB (2013). Intratumour variation of biomarker expression by immunohistochemistry in resectable non-small cell lung cancer. Eur J Cancer.

[67] Almendro V, Marusyk A, Polyak K (2013). Cellular heterogeneity and molecular evolution in cancer. Annu Rev Pathol.

[68] van der Loos CM (2010). Chromagens in multiple immunohistochemical staining used for visual assessment and spectral imaging: the colorful future. J Histotechnol.

[69] Yoshikawa H, Ehrhart EJ, Charles JB, Thamm DH, Larue SM (2012). Immunohistochemical characterization of feline oral squamous cell carcinoma. Am J Vet Res.

[70] Landini G (2013). Color deconvolution using ImageJ. http://www.mecourse.com/landinig/software/software.html.

[71] Ruifrok AC, Johnston DA (2001). Quantification of histochemical staining by color deconvolution. Anal Quant Cytol Histol.

[72] Tuominen VJ, Ruotoistenmaki S, Viitanen A, Jumppanen M, Isola J (2010). ImmunoRatio: a publicly available web application for quantitative image analysis of estrogen receptor (ER), progesterone receptor (PR), and Ki-67. Breast Cancer Res.

