# Supplementary material for: Immunohistochemical quantification of the cobalamin transport protein, cell surface receptor and Ki-67 in naturally occurring canine and feline malignant tumors and in adjacent normal tissues
Source: Oncotarget. 2014 Dec 11;6(4):2331–48. doi: 10.18632/oncotarget.3206 (PMC4385855; doi:10.18632/oncotarget.3206)
Supplement: Supplementary file 1 [file oncotarget-06-2331-s001.pdf]

Immunohistochemical quantification of the cobalamin transport protein, cell surface receptor and Ki-67 in naturally occurring canine and feline malignant tumors and in adjacent normal tissues

Supplementary Material

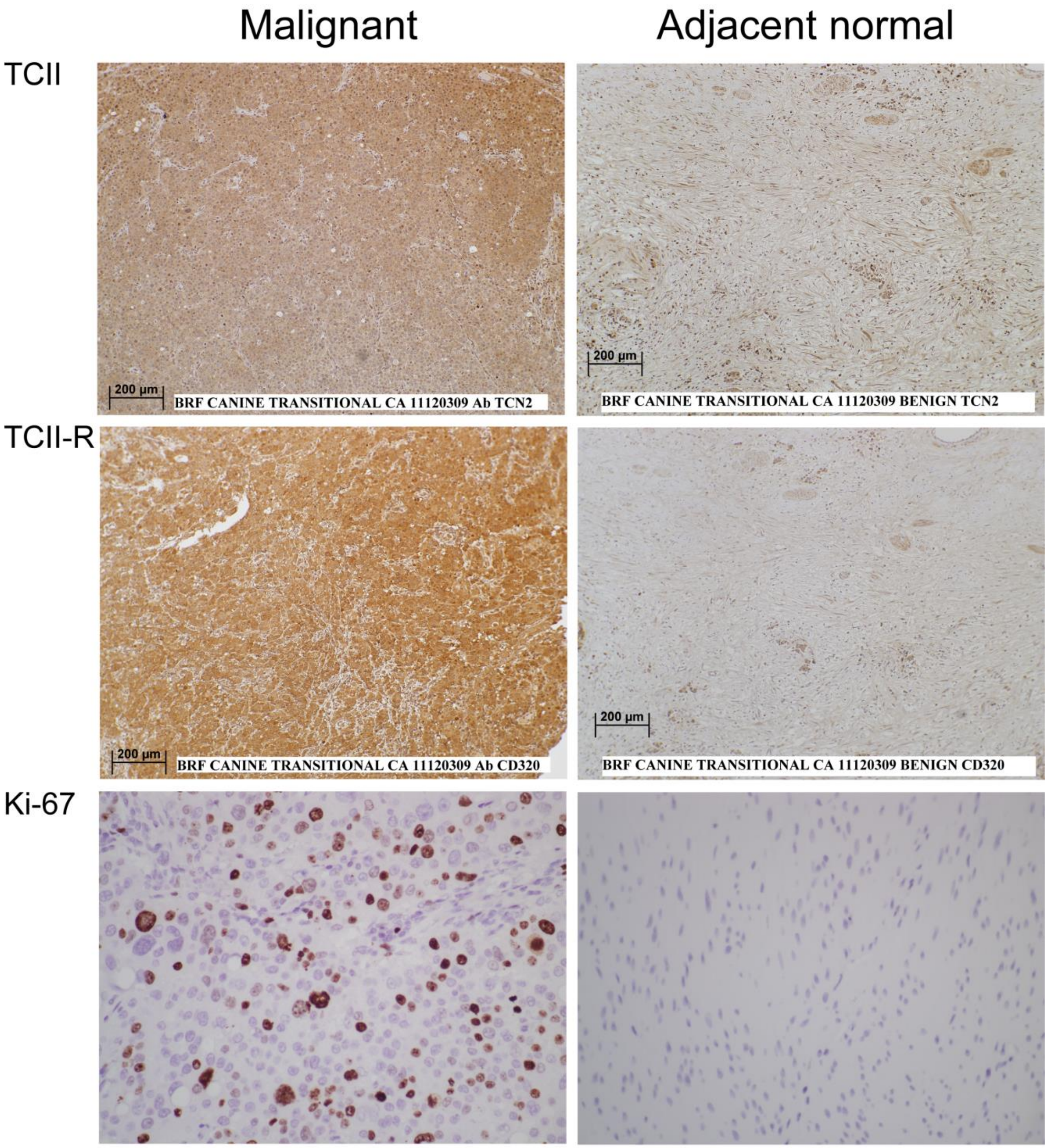

High resolution digital images of stained canine tissue sections, Case 30.

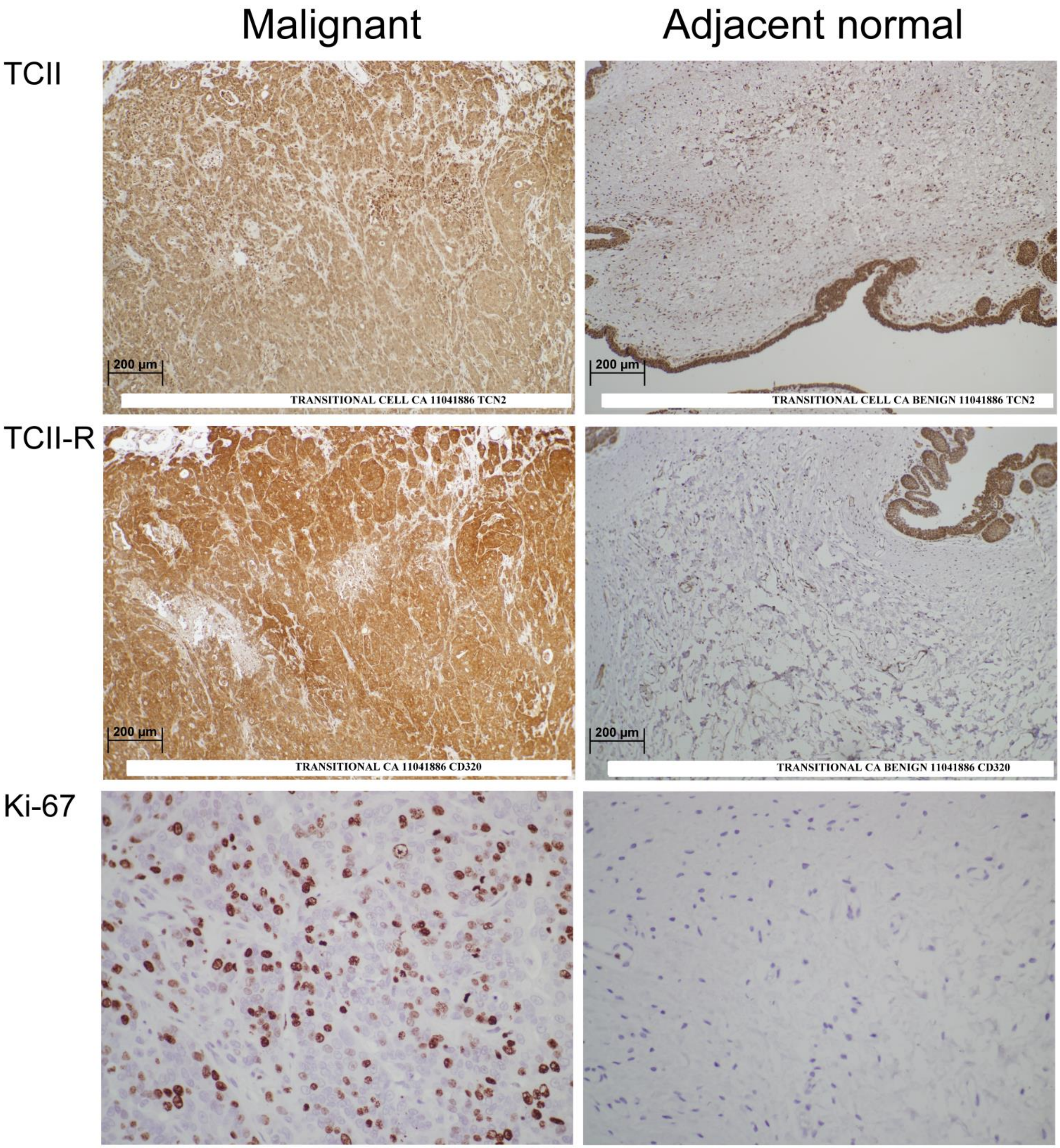

High resolution digital images of stained feline tissue sections, Case 36.
